# The predictive value of preoperative clinical laboratory indicators combined with sarcopenia for postoperative complications of gastric cancer

**DOI:** 10.3389/fmed.2026.1740026

**Published:** 2026-05-08

**Authors:** Shaoxiong Bai, Changxing Fang, Xin Zhang, Yanan Shi, Xiaojuan Tian, Tian Yao, Yu Zhang, Xiaobo Ma, Hao Chen, Yafei Cheng, Kai Jia, Jiansheng Guo, Ying Qiao, He Huang

**Affiliations:** 1 Department of Gastrointestinal Surgery, The First Hospital of Shanxi Medical University, Taiyuan, Shanxi, China; 2 Affiliated Cancer Hospital of Shanxi Medical University, Taiyuan, Shanxi, China; 3 Department of CT and Diagnostic Radiology, The First Hospital of Shanxi Medical University, Taiyuan, Shanxi, China

**Keywords:** CT, gastric tumor, postoperative complications, prediction model, sarcopenia

## Abstract

**Background:**

Postoperative complications (POCs) significantly affect recovery and prognosis in patients with gastric cancer. This retrospective cohort study aimed to develop and validate a nomogram integrating preoperative clinical laboratory indicators and sarcopenia to predict POCs in patients undergoing radical gastrectomy.

**Method:**

A retrospective analysis was conducted on 181 patients who underwent radical gastrectomy for gastric cancer between October 2019 and June 2022. Patients were randomly assigned to a training set (*n* = 126) and a validation set (*n* = 55). Sarcopenia was defined using the skeletal muscle index (SMI) measured at the third lumbar vertebra on preoperative computed tomography (CT) images. Univariate and multivariate logistic regression analyses were performed to identify risk factors for POCs (Clavien–Dindo grade ≥ II). A predictive nomogram was constructed based on independent risk factors, and model performance was assessed using the area under the receiver operating characteristic curve (AUC), calibration curves, and decision curve analysis (DCA).

**Results:**

Inter-observer consistency for SMI measurement was excellent (intraclass correlation coefficient [ICC] = 0.86). Univariate analysis revealed that clinical N stage, carcinoembryonic antigen (CEA) level, sarcopenia, and CT-assessed serosal invasion were significantly associated with POCs (all *p* < 0.05). The nomogram incorporating these four factors showed good predictive performance, with AUCs of 0.865 (95% confidence interval [CI]: 0.794–0.936) in the training set and 0.826 (95% CI: 0.704–0.948) in the validation set. Calibration curves demonstrated good agreement between predicted and observed probabilities, and DCA confirmed the model’s clinical utility.

**Conclusion:**

The nomogram integrating preoperative CEA, sarcopenia, CT-assessed serosal invasion, and clinical N stage effectively predicts the risk of postoperative complications in gastric cancer patients, facilitating preoperative risk stratification and personalized perioperative management.

## 1 Introduction

Gastric cancer is the fourth leading cause of cancer-related deaths worldwide and is one of the most common malignant tumors in Asia (1, 2). Most patients are diagnosed at an advanced stage, with poor prognosis. Currently, the main treatment method for gastric cancer patients is radical gastrectomy. However, with the aging of the population, the number of elderly patients is gradually increasing, often accompanied by higher complications. The incidence of postoperative complications (POCs) for gastric cancer is 12.5–51%, significantly affecting the prognosis of patients and reducing their overall survival rate (3, 4).

Sarcopenia is a sign of malnutrition and cachexia, characterized by reduced skeletal muscle mass, and is a quantifiable marker of weakness. In 2018, the European Working Group on Sarcopenia in the Elderly held a meeting to update the diagnostic criteria for sarcopenia: low muscle mass or quality (5–7). Studies have shown that sarcopenia not only significantly increases the risk of age-related outcomes such as fractures and falls in the elderly, but also occurs more frequently in gastric cancer patients than in the general population, and can significantly affect the disease status and survival of patients, and is of great significance for predicting the prognosis of gastric cancer (8).

This study retrospectively analyzed the clinical and pathological data of 181 gastric cancer patients admitted to our hospital from October 2019 to June 2022, and explored the predictive value of a nomogram combining preoperative clinical laboratory indicators with sarcopenia for postoperative complications of gastric cancer.

## 2 Materials and methods

### 2.1 Study design and population

This retrospective cohort study included 181 patients who underwent radical gastrectomy for gastric cancer at the First Hospital of Shanxi Medical University from October 2019 to June 2022. The cohort comprised 155 males and 26 females, with a mean age of 63 ± 10 years. Patients were randomly divided into a training set (*n* = 126) and a validation set (*n* = 55) using a 7:3 ratio. The training set was used to build the prediction model, and the validation set was used to evaluate its performance. The study was approved by the hospital’s medical ethics committee (Approval No. K-K0191), and the requirement for informed consent was waived due to its retrospective nature.

### 2.2 Inclusion and exclusion criteria

Inclusion Criteria: (1) Gastric cancer confirmed by gastric endoscopy biopsy before surgery. (2) No radiotherapy or chemotherapy before surgery. (3) Elective radical gastrectomy. (4) Abdominal CT scan performed within 2 weeks before surgery and with complete CT images at the third lumbar vertebra level. (5) Complete clinical and pathological data.

Exclusion Criteria: (1) Poor quality of CT scan images. (2) Concurrent other malignant tumors. (3) Metastasis. (4) Missing clinical and pathological data.

### 2.3 Skeletal muscle area measurement and sarcopenia definition

**Figure 1:**
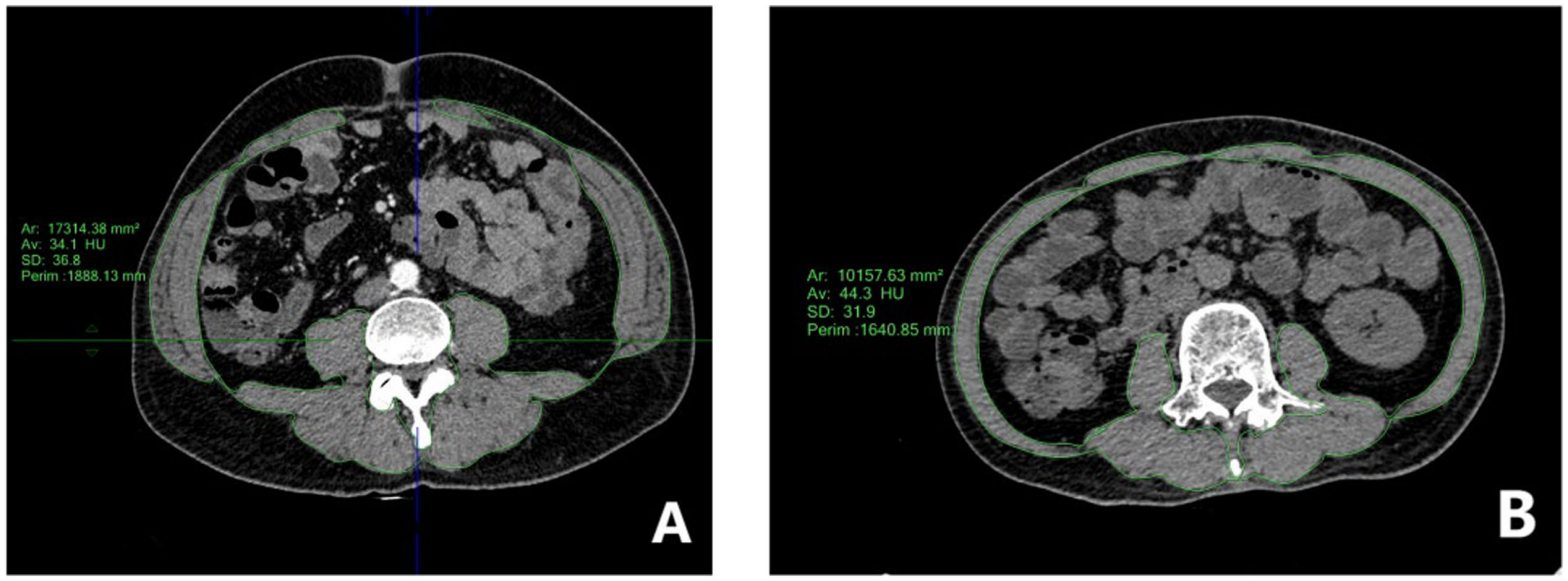
Sketch of skeletal muscle area at the L3 level of the lumbar spine in gastric cancer patients. **(A)** Screenshot of the L3 plane for non-sarcopenia gastric cancer patients, showing full muscle and thick subcutaneous fat layer; **(B)** Screenshot of the L3 plane for sarcopenia gastric cancer patients, showing thin muscle and subcutaneous fat.

The cross-sectional area of skeletal muscles at the L3 vertebral level was measured using a dedicated workstation (Philips IntelliSpace Portal) (Figure 1). The measured muscles included the psoas, paraspinal muscles (erector spinae and quadratus lumborum), and abdominal wall muscles (transversus abdominis, rectus abdominis, and internal and external obliques) (9). The skeletal muscle index (SMI, cm^2^/m^2^) was calculated by normalizing the total skeletal muscle area (cm^2^) by the square of the patient’s height (m^2^) (9).

Sarcopenia was defined using previously established sex-specific SMI cut-offs: <38.5 cm^2^/m^2^ for females and <52.4 cm^2^/m^2^ for males (7). These measurements were independently performed by a radiologist with 5 years of experience in abdominal imaging. To assess inter-observer reliability, a second radiologist with 8 years of experience randomly selected and measured 30 patients, and the intraclass correlation coefficient (ICC) was calculated.

### 2.4 Data collection and outcome definition

Clinicopathological data were collected from medical records, including age, sex, body mass index (BMI), comorbidities (hypertension, diabetes), tumor location, clinical T and N stages (based on the 8th AJCC TNM classification), Lauren classification, differentiation grade, neural invasion, vascular invasion, and preoperative laboratory indicators (albumin, CEA, CA19-9, CA72-4). CT-assessed serosal invasion and lymph node status were also recorded.

Postoperative complications (POCs) occurring within 30 days after surgery or during the hospital stay were recorded and graded according to the Clavien–Dindo classification (10, 11). Patients with complications of grade II or higher (requiring pharmacological intervention, surgical, endoscopic, or radiological intervention) were assigned to the POCs group. Patients with no complications or only grade I complications (deviation from normal postoperative course without need for intervention) were assigned to the non-POCs group.

### 2.5 Statistical analysis

Statistical analyses were performed using SPSS 26.0 and R software (version 4.2.2). Normally distributed continuous data were expressed as mean ± standard deviation and compared using the independent samples *t*-test. Non-normally distributed data were expressed as median (interquartile range) and compared using the Mann–Whitney U test. Categorical data were presented as frequencies (percentages) and compared using the *χ*^2^ test or Fisher’s exact test, as appropriate. Inter-observer reliability for SMI was assessed using the ICC, with an ICC > 0.80 considered excellent.

Univariate logistic regression analysis was performed in the training set to identify factors associated with POCs. Variables with a *p*-value < 0.05 in the univariate analysis were candidates for inclusion in a multivariate logistic regression model to identify independent risk factors. A predictive nomogram was then constructed based on the independent risk factors identified.

The predictive performance of the model was evaluated in both the training and validation sets. Discriminative ability was quantified by the area under the receiver operating characteristic curve (AUC). Calibration was assessed using calibration plots (comparing predicted vs. observed probabilities) and the Hosmer–Lemeshow test. Clinical utility was evaluated using decision curve analysis (DCA). A two-tailed *p*-value < 0.05 was considered statistically significant.

Variables with *p* < 0.05 in univariate analysis were entered into a multivariate logistic regression model using the enter method. Multicollinearity was assessed using variance inflation factors (VIFs), with all VIFs < 2 indicating no significant collinearity. No significant interaction terms were identified. Variable coding was as follows: clinical N stage was treated as an ordinal variable (N0, N1, N2, N3); CEA as dichotomous (≤5.0 ng/mL vs. >5.0 ng/mL); sarcopenia as binary (yes/no); and CT-assessed serosal invasion as binary (yes/no).

## 3 Results

### 3.1 Patient characteristics and inter-observer agreement

Among the 181 patients, 45 (24.9%) experienced POCs of grade II or higher. In the POCs group, 33 patients had grade II complications (29 with infections, 4 requiring blood transfusion or total parenteral nutrition), and 12 patients had grade III complications (4 with anastomotic leakage, 7 requiring endoscopic stent placement, 1 requiring surgical intervention for bleeding). No grade IV or V complications were observed. The remaining 136 patients (75.1%) were in the non-POCs group. The inter-observer agreement between the two physicians for assessing skeletal muscle area at the L3 vertebral level was excellent (ICC = 0.86).

### 3.2 Factors associated with postoperative complications

The comparison of clinicopathological characteristics between the POCs and non-POCs groups in both the training and validation cohorts is presented in Table 1. In the training set, significant differences were observed for CEA levels, sarcopenia, and CT-assessed serosal invasion (all *p* < 0.05). Univariate logistic regression analysis in the training set (Table 2) identified four factors significantly associated with POCs: clinical N stage (OR = 1.45, 95% CI: 1.19–2.05, *p* = 0.045), elevated CEA (>5.0 ng/mL) (OR = 1.32, 95% CI: 1.13–1.79, *p* = 0.013), presence of sarcopenia (OR = 2.73, 95% CI: 1.19–6.26, *p* = 0.018), and CT-assessed serosal invasion (OR = 4.55, 95% CI: 1.91–10.84, *p* = 0.001). In multivariate analysis, all four factors remained independent predictors (*p* < 0.05 for all).

**Table 1:** Comparison of clinicopathological characteristics between gastric cancer patients with (*n* = 45) and without (*n* = 136) postoperative complications.

Variable	Training cohort (*N* = 126)			Validation cohort (*N* = 55)		
	Non-POCs (*N* = 95)	POCs (*N* = 31)	*p* value	Non-POCs (*N* = 41)	POCs (*N* = 14)	*p* value
Age	62 ± 11	66 ± 8	0.151	64 ± 10	66 ± 7	0.415
Sex			0.969			0.051
Male	83 (87.4)	27 (87.1)		31 (75.6)	14 (100.0)	
Female	12 (12.6)	4 (12.9)		10 (24.4)	0 (0.0)	
BMI	23.3 ± 3.5	23.2 ± 3.1	0.953	22.8 ± 3.2	21.9 ± 2.2	0.346
Hypertension			0.521			0.756
Yes	25 (26.3)	10 (32.3)		10 (24.4)	4 (28.6)	
No	70 (73.7)	21 (67.7)		31 (75.6)	10 (71.4)	
Diabetes			0.851			0.242
Yes	14 (14.7)	5 (16.1)		2 (4.9)	2 (14.3)	
No	81 (85.3)	26 (83.9)		39 (95.1)	12 (85.7)	
Tumor location			0.758			0.436
Cardia	38 (40.0)	14 (45.2)		16 (39.0)	8 (57.1)	
Gastric body	13 (13.7)	5 (16.1)		7 (17.1)	1 (7.1)	
Antrum	44 (46.3)	12 (38.7)		18 (43.9)	5 (35.7)	
Clinical T stage			0.899			0.420
T1	13 (13.7)	5 (16.1)		6 (14.6)	0 (0.0)	
T2	10 (10.5)	4 (12.9)		5 (12.2)	1 (7.1)	
T3	41 (43.2)	14 (45.2)		19 (46.3)	8 (57.1)	
T4	31 (32.6)	8 (25.8)		11 (26.8)	5 (35.7)	
Clinical N stage			0.061			0.449
N0 + N1	46 (48.4)	21 (67.7)			28 (68.3)	8 (57.1)
		N2 + N3	49 (51.6)	10 (32.3)	13 (31.7)	6 (42.9)
Lauren classification			0.391			0.270
Diffuse type	22 (23.2)	9 (29.0)		11 (26.8)	2 (14.3)	
Intestinal type	55 (57.9)	13 (41.9)		24 (58.5)	12 (85.7)	
Mixed type	14 (14.7)	6 (19.4)		5 (12.2)	0 (0.0)	
Other	4 (4.2)	3 (9.7)		1 (2.4)	0 (0.0)	
Differentiation grade			0.602			0.717
Well	40 (42.1)	13 (41.9)		19 (46.3)	6 (42.9)	
Moderate	47 (49.5)	17 (54.8)		21 (51.2)	7 (50.0)	
Poor	8 (8.4)	1 (3.2)		1 (2.4)	1 (7.1)	
Neural invasion			0.291			0.559
Yes	65 (68.4)	18 (58.1)		27 (65.9)	8 (57.1)	
No	30 (31.6)	13 (41.9)		14 (34.1)	6 (42.9)	
Vascular invasion			0.135			0.705
Yes	42 (44.2)	9 (29.0)		17 (41.5)	5 (35.7)	
No	53 (55.8)	22 (71.0)		24 (58.5)	9 (64.3)	
Albumin levels			0.952			0.159
<35 g/L	71 (74.7)	23 (74.2)		29 (70.7)	7 (50.0)	
≥35 g/L	24 (25.3)	8 (25.8)		12 (29.3)	7 (50.0)	
CEA			0.011			0.050
≤5.0	79 (83.2)	19 (61.3)		34 (82.9)	8 (57.1)	
>5.0	16 (16.8)	12 (38.7)		7 (17.1)	6 (42.9)	
CA19-9			0.475			0.615
≤23	74 (77.9)	26 (83.9)		32 (78.0)	10 (71.4)	
>23	21 (22.1)	5 (16.1)		9 (22.0)	4 (28.6)	
CA72-4			0.541			0.975
≤6.9	81 (85.3)	25 (80.6)		35 (85.4)	12 (85.7)	
>6.9	14 (14.7)	6 (19.4)		6 (14.6)	2 (14.3)	
Sarcopenia			0.016			0.101
Yes	32 (33.7)	18 (58.1)		16 (39.0)	9 (64.3)	
No	63 (66.3)	13 (41.9)		25 (61.0)	5 (35.7)	
CT-assessed serosal invasion			0.001			0.037
Yes	30 (31.6)	21 (67.7)		19 (46.3)	11 (78.6)	
No	65 (68.4)	10 (32.3)		22 (53.7)	3 (24.1)	
CT-assessed lymph node status			0.159			0.119
Yes	64 (67.4)	25 (80.6)		26 (63.4)	12 (85.7)	
No	31 (32.6)	6 (19.4)		15 (36.6)	2 (14.3)	

**Table 2:** Univariate analysis of factors associated with postoperative complications in gastric cancer patients.

Variable	Univariate analysis		Multivariate analysis	
	OR (95%CI)	*p* value	OR (95%CI)	*p* value
Clinical N stage	1.45 (1.19, 2.05)	0.045	1.62 (1.21–2.17)	0.002
CEA	1.32 (1.13, 1.79)	0.013	2.92 (1.36–6.26)	0.006
Sarcopenia	2.73 (1.19, 6.26)	0.018	3.05 (1.38–6.73)	0.005
CT-assessed serosal invasion	4.55 (1.91, 10.84)	0.001	4.76 (2.02–11.19)	<0.001

### 3.3 Development and validation of the predictive nomogram

**Figure 2:**
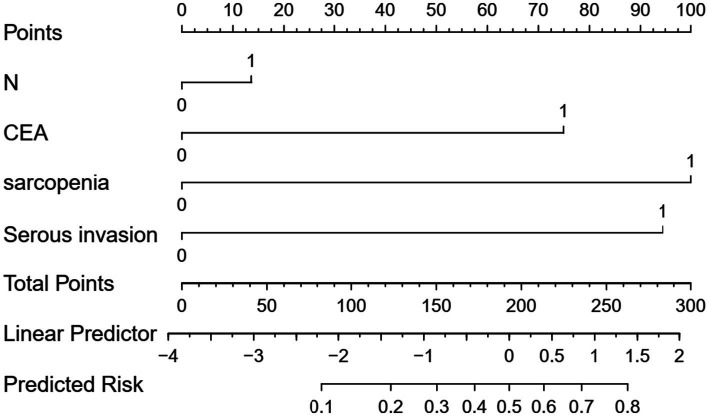
Nomogram for predicting the risk of postoperative complications in gastric cancer patients, developed from the combined model in the validation cohort.

A nomogram was constructed based on the four independent predictors (clinical N stage, CEA, sarcopenia, and CT-assessed serosal invasion) identified in the training set (Figure 2).

**Figure 3:**
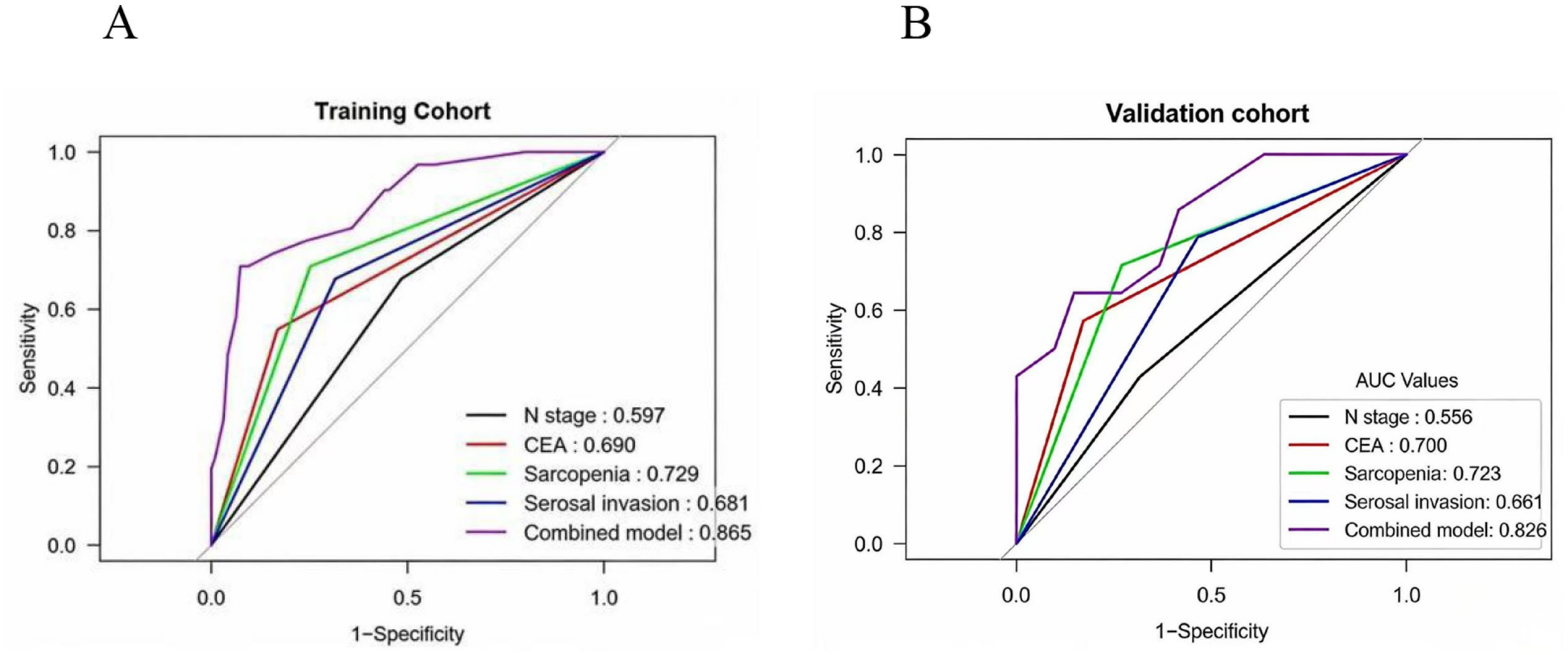
Receiver operating characteristic (ROC) curves of the predictive model in the training and validation cohorts. **(A)** Training set; **(B)** Validation set.

The predictive performance of the individual factors and the combined model was assessed using ROC curves. In the training set, the AUCs for clinical N stage, CEA, sarcopenia, and CT-assessed serosal invasion were 0.597, 0.690, 0.729, and 0.681, respectively. The combined model demonstrated superior discriminative ability, with an AUC of 0.865 (95% CI: 0.794–0.936) (Figure 3A). This was confirmed in the validation set, where the combined model achieved an AUC of 0.826 (95% CI: 0.704–0.948), compared to 0.556, 0.700, 0.723, and 0.661 for the individual factors (Figure 3B).

**Figure 4:**
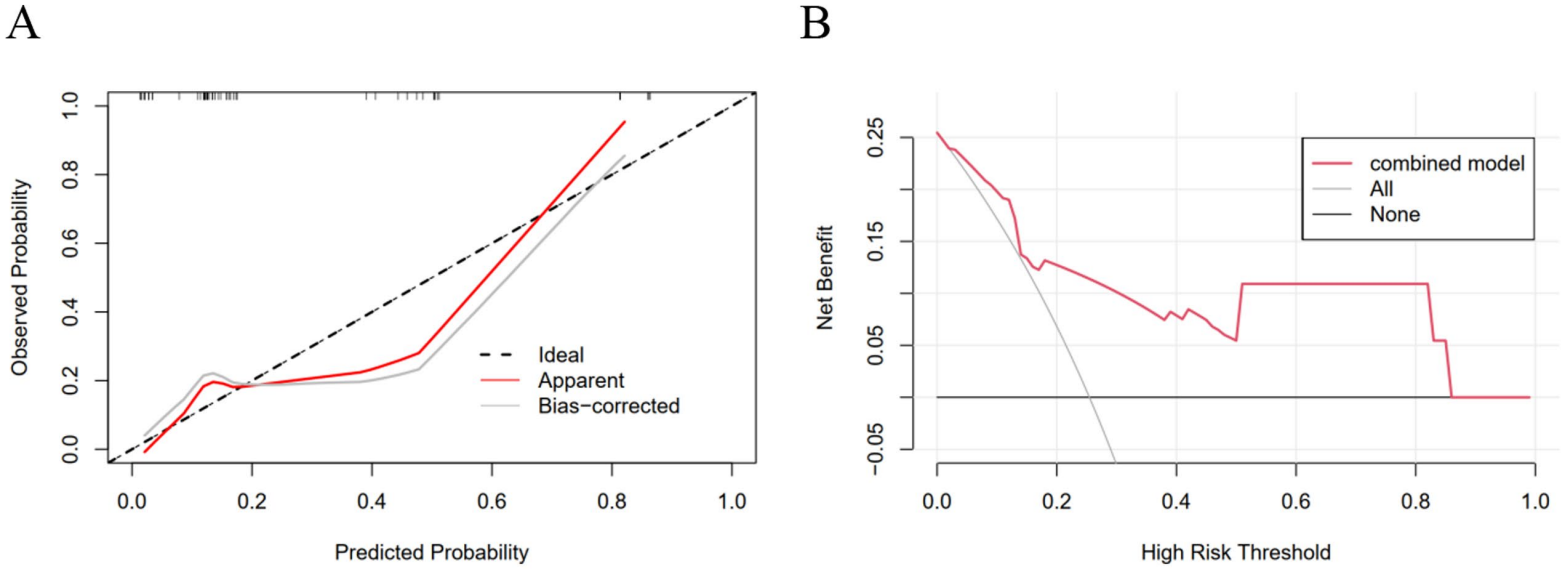
Calibration and decision curve analysis of the combined predictive model in the validation cohort. **(A)** Calibration curve in the validation cohort; **(B)** DCA in the validation set.

Calibration curves for the validation set showed good agreement between the nomogram-predicted probabilities and the actual observed outcomes (Figure 4A). Decision curve analysis (DCA) for the validation set demonstrated that the combined nomogram provided a higher net benefit across a wide range of threshold probabilities compared to a “treat-all” or “treat-none” strategy, indicating good clinical utility (Figure 4B).

## 4 Discussion

Postoperative complications not only impede physiological recovery and prolong hospital stay, thereby increasing healthcare costs, but may also exert long-term negative effects on patients’ quality of life and psychological well-being. Through systematic analysis, this study identified several significant factors associated with postoperative complications in gastric cancer patients, including higher clinical N stage, elevated preoperative carcinoembryonic antigen (CEA) levels, the presence of sarcopenia, and CT-assessed serosal invasion. Based on these four independent predictors, we developed a preoperative nomogram that provides an intuitive and quantitative individual risk score, which may assist clinicians in preoperative risk stratification and informed decision-making.

We noted an inconsistency regarding clinical N stage between the dichotomized and ordinal analyses. When clinical N stage was dichotomized as N0 + N1 versus N2 + N3, no significant difference was observed between groups (*p* = 0.061); however, when analyzed as an ordinal variable, it showed a significant association with postoperative complications (*p* = 0.045). This discrepancy likely reflects the loss of statistical power inherent in dichotomizing ordinal variables, and suggests that clinical N stage should be preserved as an ordinal predictor in future model development.

Consistent with prior research (12, 13), our study found that patients with preoperative sarcopenia had a higher likelihood of developing postoperative complications. This association may be attributed to the role of skeletal muscle as an endocrine organ, where loss of muscle mass disrupts myokine secretion, leading to reduced anti-inflammatory cytokines and increased pro-inflammatory factors (14, 15). The SMI cut-offs used in this study (38.5 cm^2^/m^2^ for females and 52.4 cm^2^/m^2^ for males) were derived from the landmark study by Prado et al. (7), which was conducted in a predominantly Western population with solid tumors. Although these thresholds have been widely adopted in oncology sarcopenia research and demonstrated significant predictive value in our Asian cohort, we acknowledge that ethnicity-specific thresholds may be more appropriate. Furthermore, the updated European consensus (EWGSOP2) emphasizes the inclusion of muscle strength and physical performance in sarcopenia diagnosis, which were not assessed in this retrospective radiology-based study.

Our results also indicated that advanced clinical N stage, elevated preoperative CEA levels, and CT-assessed serosal invasion were significantly associated with an increased risk of postoperative complications. These factors are often correlated with more aggressive tumor characteristics, such as larger tumor size, poorer differentiation, deeper invasion, and higher rates of lymph node metastasis (16, 17). Consequently, patients presenting with these features typically require more extensive surgical resection, which may contribute to higher postoperative morbidity.

In this study, we successfully developed and validated a preoperative prediction model incorporating clinical laboratory indicators and sarcopenia assessment. The model demonstrated strong discriminative ability, with an AUC of 0.865 (95% CI: 0.794–0.936) in the training set and 0.826 (95% CI: 0.704–0.948) in the validation set. This tool enables preoperative risk stratification, facilitating the identification of high-risk individuals. For such patients, a multidisciplinary team approach involving gastrointestinal surgery, nutrition support, nursing, and oncology can be implemented to deliver individualized perioperative management strategies, including preoperative nutritional optimization, meticulous surgical techniques, and tailored postoperative rehabilitation.

An important consideration is whether our preoperative prediction model can be applied to patients receiving neoadjuvant chemotherapy, which has become a standard approach for locally advanced gastric cancer. The current study exclusively included patients who underwent upfront surgery; therefore, the generalizability of our findings to the neoadjuvant setting remains uncertain. Neoadjuvant chemotherapy may influence several predictors in our model: CEA levels can fluctuate during treatment, sarcopenia may worsen due to treatment-related catabolism, and CT-assessed serosal invasion may change following tumor response. Future studies should prospectively validate and potentially recalibrate the model in cohorts receiving neoadjuvant chemotherapy, incorporating post-treatment reassessment of predictors to capture dynamic changes.

This study has several limitations. First, its single-center, retrospective design with a relatively small sample size may introduce selection bias. Second, the lack of external validation limits the generalizability of our findings. Third, sarcopenia was defined solely by radiological muscle mass using a widely adopted SMI cut-off, without incorporating measures of muscle strength or physical performance as recommended by updated consensus definitions. Additionally, the validation set was relatively small (*n* = 55, with only 14 events), which may lead to optimistic estimates of model performance. Future studies should validate our model in larger, multicenter cohorts, integrate functional parameters to better capture the multidimensional nature of sarcopenia, and explore ethnicity-specific SMI thresholds to enhance the model’s applicability across diverse populations.

In conclusion, the nomogram incorporating clinical N stage, CEA, sarcopenia, and CT-assessed serosal invasion serves as a useful tool for predicting postoperative complications and guiding personalized treatment planning in gastric cancer patients. Future work will involve applying machine learning techniques and validating the model in larger, prospective, multi-center cohorts.

## Data Availability

The data analyzed in this study is subject to the following licenses/restrictions: all datasets utilized in this study can be requested from the corresponding author. Requests to access these datasets should be directed to hh93003@163.com.
